# Clinicians’ Perspectives on Strengthening Interprofessional Teamwork to Support Surrogate Decision-Makers of Critically Ill Patients in ICUs

**DOI:** 10.1097/CCE.0000000000001365

**Published:** 2026-01-05

**Authors:** Amanda C. Moale, Vlad Razskazovskiy, Kimberly J. Rak, Aaron Richardson, Neha Dhole, Rachel A. Butler, S. Mehdi Nouraie, Maya I. Ragavan, Elizabeth A. McGuier, Douglas B. White

**Affiliations:** 1 Division of Pulmonary, Allergy, Critical Care and Sleep Medicine, Department of Medicine, University of Pittsburgh, Pittsburgh, PA.; 2 Department of Medicine, University of Pittsburgh School of Medicine, Pittsburgh, PA.; 3 Department of Critical Care Medicine, University of Pittsburgh School of Medicine, Pittsburgh, PA.; 4 Department of Medicine, West Virginia School of Medicine, Morgantown, WV.; 5 Injury Prevention Research Centre, Public Health Foundation of India, New Delhi, India.; 6 Department of Pediatrics, University of Pittsburgh School of Medicine, Pittsburgh, PA.; 7 Department of Psychiatry, University of Pittsburgh School of Medicine, Pittsburgh, PA.

**Keywords:** implementation science, interdisciplinary communication, interprofessional relations, medical decision-making, patient care planning

## Abstract

**IMPORTANCE:**

Professional societies recommend interprofessional collaboration to support ICU surrogate decision-makers, yet little is known about how to operationalize it.

**OBJECTIVES:**

Determine clinicians’ perceived acceptability of interprofessional collaboration to support surrogates of ICU patients facing goals-of-care (GOC) decisions and identify barriers/facilitators to implementing a proposed interprofessional collaboration intervention.

**DESIGN, SETTING, AND PARTICIPANTS:**

Mixed-methods study with ICU clinicians from four hospitals in Pennsylvania and Ohio. Surveys assessed acceptability across three domains: perceived effectiveness, self-efficacy, and attitudes. Clinicians from two ICUs without an interprofessional collaboration program also answered interview questions eliciting barriers/facilitators to implementing a proposed interprofessional collaboration intervention.

**ANALYSIS:**

Descriptive statistics for survey data and content analysis of interview transcripts.

**RESULTS:**

We surveyed 56 clinicians: 25 physicians and advanced practice providers (APPs), and 31 other healthcare professionals (22 nurses, 3 social workers, 6 others), and interviewed 24. Ninety-eight percent agreed that enhanced interprofessional collaboration improves surrogate support. Among other healthcare professionals, 61% wanted a larger role in GOC decisions, 97% felt confident providing emotional support, and more than 74% were confident in reinforcing prognostic information and discussing values/preferences and GOC. ICU physicians/APPs were all comfortable with nurses and social workers providing emotional support, and most were comfortable with nurses (> 80%) and social workers (> 60%) reinforcing prognostic information and discussing values/preferences, and GOC. Although more than 95% of nurses and others were confident discussing physician-proposed treatment options, only 33% of social workers were, and less than or equal to 50% ICU physicians/APPs were comfortable with nurses/social workers doing so. 94% of ICU physicians/APPs supported adopting an interprofessional collaboration intervention with shared mental models, defined roles, and training. Barriers included team turnover, time constraints, evolving care plans, and training feasibility. Facilitators included specialized training, clear roles, and knowledge of the evidence base for interprofessional collaboration.

**CONCLUSIONS:**

Most clinicians across roles found interprofessional collaboration for GOC decisions acceptable, with clear responsibilities, specialized training, workflow fit, and education on its value as key facilitators for implementation.

**KEY POINTS**

**Objective:** Determine clinicians’ perspectives on interprofessional collaboration acceptability to support surrogate decision-makers of ICU patients facing goals-of-care (GOC) decisions and identify barriers and facilitators to implementing a proposed interprofessional collaboration intervention.

**Findings:** Most clinicians found enhanced interprofessional collaboration for GOC decisions acceptable for a range of team members, and we identified key barriers and facilitators to interprofessional collaboration intervention adoption and implementation.

**Meaning:** Our findings support adapting or developing interprofessional collaboration interventions with clearly defined roles and specialized support training as core elements, and clinician education on its value and compatibility with ICU workflows as key facilitators for implementation.

Over 5 million people are admitted to an ICU in the United States annually (1). During critical illness, many patients lose decision-making capacity and must rely on surrogate decision-makers to make healthcare decisions on their behalf (2). However, communication around goals-of-care (GOC) decisions remains suboptimal (3–5), with patients commonly receiving care and treatments misaligned with their reported preferences and values (6–8).

Professional societies recommend enhanced interprofessional collaboration to support surrogates and improve communication around GOC decisions in ICUs (9). In a recent clinical trial, PARTNER (Pairing Re-engineered ICU Teams with Nurse-Driven Emotional Support and Relationship Building), a team-based approach, improved patient- and family-centeredness of care and reduced ICU length of stay for patients at high risk of death (10). However, little is known about how to operationalize interprofessional collaboration in practice, and during the trial, some clinicians expressed hesitancy about expanding non-physicians’ roles in GOC decisions in practice (10, 11). To optimize implementation, it is critical to further evaluate the acceptability of interprofessional collaboration to support surrogates during GOC decisions and better understand the key implementation determinants (12, 13).

Therefore, we sought to determine clinicians’ perspectives on the acceptability of interprofessional collaboration to support surrogate decision-makers of ICU patients facing GOC decisions, and to identify barriers and facilitators to implementing a proposed interprofessional collaboration intervention, such as PARTNER.

## MATERIALS AND METHODS

### Study Design

Data for this analysis were collected as part of a comprehensive mixed-methods study assessing ICU team collaboration practices and healthcare clinicians’ perspectives on interprofessional collaboration to support surrogate decision-makers facing GOC decisions. The parent study used cross-sectional surveys, semi-structured qualitative interviews, and ethnography across four hospitals in Pennsylvania and Ohio. Two of the included hospitals had an existing interprofessional collaboration program, based on the PARTNER intervention, in which nurses received specialized serious illness communication training and had pre-specified responsibilities for supporting surrogates (10). The other two hospitals did not have any formal interprofessional collaboration program. This article presents survey data evaluating clinicians’ acceptability of interprofessional collaboration. It also presents a subset of qualitative interview data on perceived barriers and facilitators to implementing a proposed interprofessional collaboration intervention like the PARTNER program, from participants at the two hospitals without the PARTNER program. Although the survey quantified clinicians’ acceptability of interprofessional collaboration, the qualitative data helped contextualize and further describe factors that may affect acceptability and implementation. Ethnographic data have been previously detailed and are not included in this article, nor did they inform our present analysis (14). Our reporting is guided by the Consensus-based checklist for reporting of survey studies, given the primary emphasis on our survey data (15).

### Data Collection Methods

#### Survey

There are no validated instruments for evaluating the acceptability of interprofessional collaboration to support surrogates of critically ill patients facing GOC decisions. Therefore, our study team, with expertise in critical care medicine, palliative medicine, decision-making science, implementation science, and mixed-methods research, developed a survey informed by an extensive literature review and insights from the PARTNER trial (10). Our survey (**Supplement 1**, https://links.lww.com/CCX/B593) included five-point Likert-scale items, binary yes-no items, and open-ended responses. In accordance with the theoretical framework of the study by Sekhon et al (16)for acceptability, our survey evaluated three key acceptability domains related to interprofessional collaboration: perceived effectiveness, self-efficacy, and attitudes. Specifically, we assessed clinicians’ perceived effectiveness of interprofessional collaboration (items 3.3 and 4.2); other healthcare professionals’ (i.e., nurses, social workers, dieticians, spiritual care, and the care manager) self-efficacy in working with physicians/APPs to perform specific support tasks and their desire to take on a larger role (items 5.1, 5.2, and 5.3); and ICU physicians’/APPs’ attitudes toward nurses and social workers participating in those support tasks (items 4.4, 4.6, and 4.7). We evaluated ICU physicians’/APPs’ attitudes both at baseline (item 4.4) and after presenting a prompt describing a hypothetical interprofessional collaboration intervention that included specialized clinician training, a shared mental model among team members, and strategies to build trust (items 4.5, 4.6, and 4.7).

#### Interviews

We developed interview guides based on literature review, relevant constructs from Organizational Theory and Behavior (17, 18), and the expertise of the study team. The guides covered four key topics: 1) how participants and the ICU team currently provide decision support to surrogates; 2) typical processes of family conferences; 3) ICU team dynamics in supporting surrogates; and 4) overall thoughts on structuring support. This article presents data from a subset of questions asked of clinicians at sites without the PARTNER program, focused on perceived barriers and facilitators to implementing a proposed interprofessional collaboration intervention like the PARTNER program (**Supplement 2**, https://links.lww.com/CCX/B593).

Both the survey and interview guides were iteratively revised by our study team for clarity, relevance, completeness, and optimal length before administration. The interviews in the parent mixed-method study were conducted until thematic saturation was reached at each hospital, that is, the point at which no new codes or themes were generated across the four key topics described earlier (19).

### Sample Characteristics and Administration

We purposefully recruited multidisciplinary clinicians from four different hospitals in Pennsylvania and Ohio to participate in a semi-structured qualitative interview followed by the survey. One ICU per hospital was included, comprising two medical and two medical-surgical units. The two hospitals with the PARTNER program were academic medical centers. Of the two hospitals without PARTNER, one was a community hospital and the other an academic hospital, purposefully selected for its more racially and socioeconomically diverse patient population. Participants included physicians/APPs, nurses, social workers, care managers, spiritual care clinicians, and dieticians—selected based on ICU team members’ input regarding which professionals may support families during GOC decisions. Eligibility criteria included fluency in English and greater than or equal to 18 years. Recruitment was conducted via email and in person, with up to three outreach attempts per potential participant. Interviews and surveys were conducted during the following time frames by site: ICU1 (March 2021 to January 2022), ICU2 (May 2021 to February 2022), ICU3 (April 2022 to November 2022), and ICU4 (October 2023 to December 2023). We aimed to enroll multiple individuals within each professional role; however, some roles had only one eligible representative per ICU. Longer enrollment windows reflected multiple attempts to include individuals from less-represented roles.

Surveys were administered electronically using Qualtrics (Qualtrics, Seattle, WA) and employed branching logic to present different sets of questions based on participants’ self-identified professional role (e.g., ICU physicians/APPs, nurses, social workers, etc). All qualitative data were collected and analyzed by individuals with advanced training or experience in qualitative methodology and/or medical anthropology, with individual contributors denoted by their initials. Audio-recorded interviews were conducted by K.J.R. or A.R. by phone or in person based on participant preference. All interviews were transcribed verbatim. Surveys and interview transcripts were anonymized by removing identifiable information. This study was reviewed and approved by the University of Pittsburgh Institutional Review Board (STUDY19080333: *Improving Surrogate Decision Making for Patients with Advanced Respiratory Failure*), with initial approval granted on December 4, 2020. All participants provided verbal informed consent before data collection. The study was conducted in accordance with the ethical standards of the University of Pittsburgh Institutional Review Board and with the Helsinki Declaration of 1975.

### Statistical Analysis of Survey Data

Survey data were analyzed using descriptive statistics. Likert-scale item responses were treated as ordinal. For items ranging from “strongly agree” to “strongly disagree,” responses of “strongly agree” and “agree” were combined and recoded as “agree.” Similarly, for items ranging from “very comfortable” to “very uncomfortable,” “very comfortable” and “comfortable” were recoded as “comfortable.” For item 4.6 (5-point scale from “not supportive” to “extremely supportive”), responses from moderately to extremely supportive were recoded as “supportive.” We summarized Likert-scale responses by reporting the percentage of respondents endorsing “agree” or “comfortable,” along with medians and interquartile ranges. We summarized binary responses by reporting the percentage of respondents selecting “yes.” We excluded responses that were erroneously collected due to branching logic errors. We performed all analyses in STATA 18 (StataCorp LLC, College Station, TX).

### Content Analysis of Qualitative Data

We performed a content analysis of responses to the subset of interview questions relevant to our research question (Supplement 2, https://links.lww.com/CCX/B593) (20, 21). These analyses were conducted independently from the qualitative analyses in the parent study. Specifically, K.J.R. and either N.D. or A.R. independently performed question-based coding, labeling responses by interview question, and categorizing each response as a potential barrier or facilitator to implementing a program like PARTNER in non-PARTNER ICUs. A.C.M., who was not involved in the initial coding, reviewed the coded data and planned to adjudicate any major discrepancies; however, none arose. A.C.M. then further organized the identified barriers and facilitators by the Consolidated Framework for Implementation Research (CFIR) domain and construct, with iterative revision by the study team. Coders used NVivo software (Lumivero, Version 13) for qualitative data management (22).

## RESULTS

### Respondent and Participant Characteristics

We enrolled 56 participants (**Table 1**) from four hospitals, two with the PARTNER program and two without. Participants included 25 physicians and APPs (18 critical care and 7 palliative), 22 nurses, 3 social workers, and 6 other clinicians (1 care manager, 2 dieticians, and 3 spiritual care). We collectively refer to nurses, social workers, and other clinicians as ‘other healthcare professionals’ throughout the article. The median participant age was 38 (interquartile range, 31–47), and most participants identified as female (72.7%) and White (92.8%). Only 39.3% had prior communication training. Of the 56 participants, 24 clinicians from the hospitals without the PARTNER program also participated in the subset of interview questions about implementing a proposed interprofessional collaboration intervention like the PARTNER program. Refer to **eTable 1** (https://links.lww.com/CCX/B593) for a summary of general survey responses and **eTable 2** (https://links.lww.com/CCX/B593) for demographics by clinician role.

**TABLE 1.** Demographic Characteristics of Participating Clinicians From Four Hospitals (*n* = 56)

Age, median (interquartile range), yr^a^	38 (31–47)
Sex, *n* (%)^a^	
Female	40 (72.7)
Male	15 (27.3)
Race, *n* (%)^a^	
White	51 (92.8)
Asian	2 (3.6)
Decline to answer	2 (3.6)
Role in ICU, *n* (%)	
Attending ICU physician	12 (21.4)
ICU APPs	6 (10.7)
Palliative care attending/APP	7 (12.5)
Nurse	22 (39.3)
Social worker	3 (5.4)
Others^b^	6 (10.7)
Years in attending ICU role since training, *n* (%)	
1–5	3 (25)
6–10	3 (25)
> 10	6 (50)
Prior communication training, *n* (%)	
Yes	22 (39.3)
No	34 (60.7)

APP = advanced practice providers.

a Data missing from one participant.

b Includes one care manager, two dieticians, and three spiritual care clinicians.

### Perceived Effectiveness of Interprofessional Collaboration

Nearly all participants (98.2%, *n* = 55/56) agreed that higher levels of interprofessional collaboration would improve support for surrogates facing GOC decisions. Participants cited nurses, social workers, care managers, chaplains, respiratory therapists, and palliative care clinicians as professionals whose involvement they wished to see increased.

### Self-Efficacy and Attitudes Related to Interprofessional Collaboration

Most other healthcare professionals (93.5%, *n* = 29/31) felt they played an important role in supporting surrogates facing GOC decisions, and 61.3% expressed a desire to take on a larger role. Nearly all (96.8%) were interested in further communication training for supporting surrogates facing GOC decisions.

**Table 2** displays other healthcare professionals’ confidence in working with physicians/APPs to perform specific support tasks, as well as ICU physicians’/APPs’ comfort with nurses and social workers performing support tasks. All nurses and social workers (100%, *n* = 25) reported confidence in providing emotional support to surrogates, and all ICU physicians/APPs (100%, *n* = 16) were comfortable with them doing so. Additionally, most other clinicians (83.3%, *n* = 5/6) were confident in providing emotional support. Most nurses (> 75%), social workers (66.7%), and other clinicians (≥ 66.7%) were also confident in their ability to reinforce prognostic information provided by physicians and to discuss patients’ values, preferences, and overall GOC. Likewise, most ICU physicians/APPs were comfortable with nurses (> 80%) and social workers (> 60%) participating in these tasks. However, although nearly all nurses (95.5%) and all other clinicians (100%) reported confidence in discussing physician-proposed treatment options, only one-third of social workers did, and just 50% of ICU physicians/APPs were comfortable with nurses and 31.3% with social workers participating in this support task.

**TABLE 2.** Other Healthcare Professionals’ Confidence (% [Median, Interquartile Range]) in Supporting Surrogates, and ICU Physicians'/Advanced Practice Providers’ Comfort With Nurses and Social Workers’ Involvement, at Baseline and After Introduction of a Hypothetical Interprofessional Collaboration Intervention

Support Task	Nurses’ Confidence (*n* = 22)	Social Workers’ Confidence (*n* = 3)	Others’^a^ Confidence (*n* = 6)	Total (Other Healthcare Professionals, *n* = 31)	ICU Physician/Advanced Practice Provider Comfort With Nurses and Social Workers Before and After Hypothetical Intervention (*n* = 16–17)^b^			
					Nurses		Social Workers	
					Before	After	Before	After
Reinforcing prognostic information	95.5% (4, 4–5)	66.7% (4, 2–4)	100% (4, 4–5)	93.5% (4, 4–5)	81.3% (4, 4–4)	93.8% (4, 4–5)	62.5% (4, 3–4)	76.5% (4, 4–5)^c^
Talking with surrogate decision-makers about physician-proposed treatment options	95.5% (4, 4–5)	33.3% (3, 2–4)	100% (5, 4–5)	90.3% (4, 4–5)	50.0% (4, 2–4)	75.0% (4, 4–5)	31.3% (3, 2–4)	50% (4, 3–4)
Talking with surrogate decision-makers about patients’ values and preferences	90.9% (4, 4–5)	66.7% (4, 2–5)	100% (5, 4–5)	90.3% (4, 4–5)	100.0% (5, 4–5)^c^	100.0% (4, 4–5)^c^	88.2% (5, 4–5)^c^	82.4% (4, 4–5)^c^
Talking with surrogate decision-makers about overall goal of care	76.2% (4, 4–5)	66.7% (4, 3–4)	66.7% (5, 3–5)	74.2% (4, 3–5)	81.3% (4, 4–5)	94.1% (4, 4–5)^c^	75.0% (4, 4–5)	76.5% (4, 4–5)^c^
Providing emotional support to surrogates	100.0% (4, 4–5)	100.0% (4, 4–5)	83.3% (5, 4–5)	96.8% (4, 4–5)	100.0% (5, 5–5)	100.0% (5, 5–5)	100.0% (5, 5–5)	93.8% (5, 5–5)

a Others included one care manager, two dieticians, and three spiritual care clinicians.

b Missing data from one ICU physician/advanced practice provider (APP), and one other ICU physician/APP only completed a subset of items due to a branching logic error, resulting in *n* = 16 or 17, depending on the item.

c Items with *n* = 17.

After introducing the hypothetical interprofessional collaboration intervention, which included specialized clinician training, a shared mental model among team members, and trust-building strategies, 93.8% (*n* = 15/16) of ICU physicians/APPs were at least moderately supportive of adopting the hypothetical interprofessional collaboration intervention. A higher percentage also reported being comfortable with nurses and social workers participating in most informational support tasks (Table 2, columns 7 and 9). The exceptions were slightly lower comfort levels with social workers discussing patients’ values and preferences (82.4%) and providing emotional support (93.8%). Although discussing physician-proposed treatment options remained the area of lowest comfort—75% for nurses and 50% for social workers—a higher percentage of clinicians were comfortable with both groups participating in this task after the hypothetical interprofessional collaboration intervention. The magnitude of change was large for several support tasks, but the differences were not statistically significant (*p* > 0.05)

### Barriers and Facilitators

Although the survey data quantified clinicians’ acceptability of interprofessional collaboration, the qualitative data offered deeper insights into perceived challenges, as well as factors influencing acceptability and implementation. **eTable 3** (https://links.lww.com/CCX/B593) summarizes clinicians’ perceived barriers and facilitators to implementing a program like PARTNER, organized by CFIR domain and construct, with representative quotes. Barriers were primarily related to ICU work infrastructure, including team turnover, time constraints, and the dynamic nature of care planning in the ICU, highlighting the importance of workflow fit. Facilitators included specialized support training for clinicians, clear role definition with consistent messaging, and education on the effectiveness of interprofessional collaboration, which was viewed as important for clinician buy-in. Notably, participants described both structured training and role clarity with aligned messaging not merely as facilitators, but as essential elements of an interprofessional collaboration intervention. Although structured training was widely regarded as critical to successful implementation, some participants raised concerns about the feasibility of integrating training into existing workflows. Finally, a few clinicians suggested that creating a dedicated support role, rather than expanding responsibilities among existing team members, may facilitate adoption of an interprofessional collaboration program like PARTNER and help overcome barriers such as team turnover and time constraints, if funding permits.

In addition to barriers and facilitators, clinicians across roles broadly supported enhanced interprofessional collaboration as a valuable and acceptable approach, reinforcing findings from the survey. Participants cited potential benefits for both the care team and surrogates/patients. As one nurse stated, “This would be beneficial to both the care team and the surrogates.”

## DISCUSSION

In this multicenter, mixed-methods study, we found that enhanced interprofessional collaboration to support surrogates facing GOC decisions was broadly acceptable to clinicians, and we identified key barriers and facilitators that may inform the implementation of interprofessional collaboration interventions in ICU practice. Given the broad acceptability, our findings support adapting or developing interprofessional collaboration interventions to support surrogates in ICUs. Clearly defined roles and specialized support training are likely core intervention elements, with clinician education on interprofessional collaboration and thoughtful integration with existing workflows likely serving as critical facilitators for effective implementation.

Prior work, such as that by You et al (23), found general clinician support for involving a range of team members in GOC discussions among seriously ill hospitalized patients. Our study extends these findings specifically to the ICU setting and expands on them by examining clinicians’ perceptions across three acceptability domains, identifying key factors that influence implementation, and offering practical insights for operationalizing interprofessional collaboration in practice—critical steps in pre-implementation that remained unexplored. However, prior trials and literature have also documented clinician hesitation around implementing interprofessional collaboration in practice (10, 23, 24). Our findings help elucidate which specific support tasks elicit hesitation. We found that tasks such as providing emotional support, reinforcing prognostic information, and discussing patients’ values, preferences, and overall GOCs were widely accepted by a range of clinicians as appropriate for various team members to perform. In contrast, social workers expressed hesitation about discussing physician-proposed treatment options, and ICU physicians/APPs were hesitant about nurses and social workers performing this task.

Our findings also build on existing literature by highlighting the potential challenges related to hierarchy, power-sharing, and role ambiguity when multiple team members are involved in supporting surrogates in ICUs (24, 25). Nevertheless, most interprofessional clinicians wanted a larger role in GOC decisions, and our findings point to potential strategies to overcome these challenges, such as by establishing clear roles and responsibilities.

Furthermore, some implementation determinants appear consistent across settings. For example, in primary care, Légaré et al (26) identified training in interprofessional collaboration and shared decision-making as common facilitators. Similarly, we found high levels of support when interprofessional collaboration was delivered through a structured intervention that included specialized clinician training and attended to key domains of effective teamwork. Notably, even the proportion of clinicians reporting comfort or confidence in interprofessional team members discussing physician-proposed treatment increased in this context.

Our qualitative data further emphasized the need for shared agreement on team members’ roles and tasks, as well as specialized support training for clinicians, suggesting that these are not only facilitators but core intervention elements. Core elements are defined as those necessary for effective practice and linked to improved outcomes (27). However, because most barriers were related to work infrastructure, a one-size-fits-all approach is unlikely to succeed across settings. Tailored strategies will likely be necessary to address context-specific barriers and optimize fit across different hospitals. For example, although some participants suggested creating a dedicated surrogate support role to address time constraints, not all hospitals will have the capacity to do so. Alternatively, time-related challenges may be overcome by clearly defined roles and thoughtful integration into the existing ICU workflow. Lastly, our findings apply to ICUs with and without existing interprofessional support programs, offering guidance for adapting or developing interprofessional collaboration interventions/programs in practice.

Our study has several strengths. It was conducted across multiple hospitals, including both academic and community settings. Additionally, to our knowledge, this is the first study to assess both overall and task-specific acceptability of interprofessional collaboration for GOC decisions in the ICU and identify the key barriers and facilitators from a range of interprofessional team members.

Our study also has several limitations. Although we included 56 participants, most were physicians and nurses, with lower representation from other roles such as social work and case management. Despite recruiting from four ICUs across different hospitals, some roles only had one eligible representative per unit, and not all responded to outreach. Furthermore, because the survey employed branching logic to assign different questions based on participants’ self-identified role, sample sizes—and thus statistical power—were limited for certain question sets. For example, although reported acceptability for several support tasks increased after introduction of the hypothetical interprofessional collaboration intervention, these differences were not statistically significant and may have reached significance with a larger sample. Additionally, perspectives on the hypothetical interprofessional collaboration intervention may have been influenced by social desirability bias, since participants were responding to a proposed scenario rather than actual practice. Finally, although the four hospitals included both academic and community settings, only one served a more racially and socioeconomically diverse patient population. Similarly, the clinician sample lacked racial diversity and was predominantly female, including for physicians and APPs (54.2% female in our sample as compared with national estimates of 28% for ICU physicians in the U.S. critical care workforce) (28). This overrepresentation may reflect selection bias and could influence perspectives on interprofessional collaboration.

Future research should aim for even greater inclusion of other healthcare professionals—including respiratory therapists, social workers, and care managers. Additionally, a focused study exploring surrogates’ perspectives on interprofessional collaboration for GOC decisions is warranted, as their insights are also essential to implementing interprofessional collaboration interventions that effectively support them. Transferability may also be augmented by expanding recruitment to more demographically and geographically diverse clinical settings, with physician/APP gender representation more closely aligned with that of the U.S. critical care medicine workforce.

## CONCLUSIONS

Among a range of interprofessional clinicians, enhanced interprofessional collaboration for GOC decisions was widely accepted, particularly when grounded in best practices of teamwork. Our findings suggest readiness to implement interprofessional collaboration interventions that include shared agreement on roles and tasks, as well as specialized training for clinicians as core components. Tailored strategies to address context-specific workflow barriers will also be essential to support successful adoption.

## Supplementary Material
