# Supplementary material for: Clinicians’ Perspectives on Strengthening Interprofessional Teamwork to Support Surrogate Decision-Makers of Critically Ill Patients in ICUs
Source: Crit Care Explor. 2026 Jan 5;8(1):e1365. doi: 10.1097/CCE.0000000000001365 (PMC12772474; doi:10.1097/CCE.0000000000001365)
Supplement: Supplementary file 1 [file cc9-8-e1365-s001.pdf]

Supplemental Content for "Clinicians' perspectives on strengthening interprofessional teamwork to support surrogate decision makers of critically ill patients in ICUs"

Amanda C. Moale, MD[1]; Vlad Razskazovskiy, MD[2]; Kimberly J. Rak, PhD, MPH[3]; Aaron Richardson, MS[4]; Neha Dhole, PhD[5]; Rachel A. Butler, MHA, MPH[3]; S. Mehdi Nouraie, MD, PhD[1]; Maya I. Ragavan, MD, MPH[6]; MS; Elizabeth A. McGuier, PhD[7]; Douglas B. White, MD, MAS[3].

[1]Division of Pulmonary, Allergy, Critical Care and Sleep Medicine, Department of Medicine, University of Pittsburgh, Pittsburgh, PA, United States

[2]Department of Medicine, University of Pittsburgh School of Medicine, Pittsburgh, PA, United States

[3]Department of Critical Care Medicine, University of Pittsburgh School of Medicine, Pittsburgh, PA, United States

[4]Department of Medicine, West Virginia School of Medicine, Morgantown, West Virginia, United States

[5]Public Health Foundation of India, New Delhi, India

[6]Department of Pediatrics, University of Pittsburgh School of Medicine, Pittsburgh, PA, United States

[7]Department of Psychiatry, University of Pittsburgh School of Medicine, Pittsburgh, PA, United States

Table of contents

Supplement 1: Clinician Survey	Pages 3-23
Supplement 2: Subset of Interview Questions Asked to Participants at Sites Without the PARNTER Program	Page 24
eTable 1. General survey questions and responses	Pages 25-28
eTable 2. Demographics by clinician role	Page 29
eTable3. Clinicians’ perceived barriers and facilitators to enhanced interprofessional support through specialty nurse training and care pathways	Page 30-31

Supplement 1: Clinician Survey

Start of Block: Demographics

1.1 Participant ID: (leave blank if not provided)

____

1.2 Do you work at a the MetroHealth System?

- Yes (1)
- No (2)

1.3 What is your role in the ICU?

- Attending (1)
- Fellow (2)
- Resident (3)
- APP (Physician's Assistant/Nurse Practitioner) (4)
- Nurse (5)
- Social Worker (6)
- Care Manager (7)
- Spiritual Care (8)
- Other: (9) ____

Display This Question:

If What is your role in the ICU? = Attending

Or What is your role in the ICU? = Fellow

Or What is your role in the ICU? = Resident

1.3.1 What specialty or subspecialty training have you had? (select all that apply)

- ▢ Pulmonary Medicine and/or Critical Care Medicine (1)
- ▢ Internal Medicine (4)
- ▢ Family medicine (5)
- ▢ Anesthesia (6)
- ▢ Neurology (7)
- ▢ Surgery (8)
- ▢ Other (Please list other specialty): (9) ____

1.4 What is your degree(s)?

____

Display This Question:

If What is your role in the ICU? = Attending

1.5 How many years have you worked in this role since completing your clinical training (e.g. terminal fellowship / residency)?

▼ Less than 1 year (1) ... 50 (51)

Display This Question:

If What is your role in the ICU? != Attending

And What is your role in the ICU? != Fellow

And What is your role in the ICU? != Resident

1.6 How many years have you worked in this role?

▼ Less than 1 year (1) ... 50 (51)

1.7 How many years have you worked at this ICU?

▼ Less than 1 year (1) ... 50 (51)

1.8 Have you ever received any additional training related to communicating with patients' families or surrogate decision makers?

○ No (9)

○ Yes (Please describe the training you received): (8)
____

1.9 What is your age?

▼ 18 (1) ... 80 (79)

End of Block: Demographics

Start of Block: General Survey Questions

3.1 Thank you for assisting us with this project. There are several important definitions to keep in mind as you complete the survey:

1. **Supporting surrogates**: providing assistance with the emotional, informational, and psychological challenges of making treatment decisions for an incapacitated patient.
2. **Goals of care decisions**: For the purposes of this survey, we are referring to decisions

about whether to initiate or continue life-prolonging treatments, or whether to transition to a comfort-focused care plan.

3. **Advanced practice providers**: Nurse practitioners and physician assistants.

3.2 Please indicate how much you agree with the following statements. In general, the healthcare team in my ICU:

	Strongly disagree (14)	Disagree (15)	Neutral (16)	Agree (17)	Strongly agree (18)
Establishes a high level of trust with surrogate decision-makers facing goal of care decisions. (6)	o	o	o	o	o
Effectively helps surrogates understand patient medical condition(s). (7)	o	o	o	o	o
Effectively helps surrogates understand patient prognoses (e.g. the likely outcomes of treatment). (8)	o	o	o	o	o
Effectively helps surrogates understand available treatment options as it relates to overall options in terms of goal of care (e.g., full life support, time-limited trial, and comfort-focused	o	o	o	o	o

care). (9)					
Effectively explores patient values and preferences as they relate to medical care. (10)	○	○	○	○	○
Effectively supports surrogates in applying patient values and preferences to decisions about overall goal of care. (11)	○	○	○	○	○
Promotes interprofessional collaboration between healthcare professions to support surrogate decision makers facing goal of care decisions. (12)	○	○	○	○	○
Achieves a high level of interprofessional collaboration between healthcare professions to support surrogate decision makers facing goal of care decisions. (13)	○	○	○	○	○

3.3 Higher levels of interprofessional collaboration between healthcare providers improves overall support provided to surrogate decision makers facing goal of care decisions.

- Strongly disagree (4)
- Disagree (5)
- Neutral (6)
- Agree (7)
- Strongly agree (8)

End of Block: General Survey Questions

Start of Block: For Physicians / APPs only

4.1 Are there any members of the ICU team that you would like to see have a higher degree of involvement in supporting surrogates facing goal of care decisions?

- Yes (1)
- No (2)

Display This Question:

If Are there any members of the ICU team that you would like to see have a higher degree of involvem... = Yes

4.2 Please select those professions that you would like to see have a higher degree of involvement in supporting surrogates facing goal of care decisions

- Registered Nurse (1)
- Social Worker (2)
- Care Manager (3)
- Chaplain (4)

☐ Other (5) ____

Display This Question:

If Are there any members of the ICU team that you would like to see have a higher degree of involvem... = Yes

4.3 Please elaborate on **why you selected** this as a profession that you would like to see have a higher degree of involvement in supporting surrogates during goal of care decisions as well as **what role** you would like to see them play.

Display This Choice:

If Please select those professions that you would like to see have a higher degree of involvement in... = Registered Nurse

☐ Registered Nurse (1) ____

Display This Choice:

If Please select those professions that you would like to see have a higher degree of involvement in... = Social Worker

☐ Social Worker (2) ____

Display This Choice:

If Please select those professions that you would like to see have a higher degree of involvement in... = Care Manager

☐ Care Manager (3) ____

Display This Choice:

If Please select those professions that you would like to see have a higher degree of involvement in... = Chaplain

☐ Chaplain (4) ____

Display This Choice:

If Please select those professions that you would like to see have a higher degree of involvement in... = Other

☐ Other (5) ____

4.4 Currently, how comfortable would you be allowing:

	Very Uncomfortable (4)	Uncomfortable (5)	Neutral (6)	Comfortable (7)	Very Comfortable (8)
Nurses to reinforce the prognostic information you provided to families (they would not deliver new information, only restate what you conveyed)? (4)	○	○	○	○	○
Nurses to talk with surrogate decision-makers about the various treatment options that the physician proposed? (17)	○	○	○	○	○
Nurses to talk with surrogate decision-makers about patients' values and preferences? (10)	○	○	○	○	○
Nurses to talk with surrogate decision-makers about overall goal of care? (13)	○	○	○	○	○

Nurses to provide emotional support to surrogates? (5)	○	○	○	○	○
Social workers to reinforce the prognostic information you provided to families (they would not deliver new information, only restate what you conveyed)? (18)	○	○	○	○	○
Social workers to talk with surrogate decision-makers about the various treatment options that the physician proposed? (11)	○	○	○	○	○
Social workers to talk with surrogate decision-makers about patients' values and preferences? (14)	○	○	○	○	○
Social workers to talk with surrogate decision-	○	○	○	○	○

makers about overall goal of care? (19)					
Social workers to provide emotional support to surrogates? (20)	○	○	○	○	○

4.5 We are considering developing an intervention that leverages multiple members of the ICU team (e.g., physicians/APPs, nurses, and social workers) to support family members making goals-of-care decisions for incapacitated, critically ill patients. The intervention would involve achieving shared agreement concerning who will support surrogates, what their respective roles will be, and how team members will work together in a well-integrated way. Additionally, the intervention would entail efforts to ensure that each team member has the needed expertise to effectively function as part of the team, and that there is trust and a shared mental model amongst all team members. Physicians and APPs would remain responsible for conveying prognostic information to surrogates, determining what are medically appropriate treatments to offer, and making treatment recommendations.

4.6 If such an intervention were developed and shown to be effective, how supportive would you be of it being adopted in your ICU?

○ Not supportive (26)

○ Slightly supportive (27)

○ Moderately supportive (28)

○ Very supportive (29)

○ Extremely supportive (30)

4.7 If members of your ICU team participated in this intervention to enhance interprofessional support to surrogates facing goals of care decisions, how comfortable would you be:

	Very Uncomfortable (4)	Uncomfortable (5)	Neutral (6)	Comfortable (7)	Very Comfortable (8)
Having trained nurses reinforce the prognostic information you provided to families (they would not deliver new information, only restate what you conveyed)? (4)	○	○	○	○	○
Allowing trained nurses to talk with surrogate decision-makers about the various treatment options that the physician proposed? (5)	○	○	○	○	○
Allowing trained nurses to talk with surrogate decision-makers about patients' values and preferences? (6)	○	○	○	○	○
Allowing trained nurses to talk with surrogate	○	○	○	○	○

decision-makers about overall goal of care? (10)					
Allowing trained nurses to provide emotional support to surrogates? (9)	○	○	○	○	○
Having trained social workers reinforce the prognostic information you provided to families (they would not deliver new information, only restate what you conveyed)? (14)	○	○	○	○	○
Allowing trained social workers to talk with surrogate decision-makers about the various treatment options that the physician proposed? (13)	○	○	○	○	○
Allowing trained social workers to talk with surrogate decision-makers about	○	○	○	○	○

patients' values and preferences? (12)					
Allowing trained social workers to talk with surrogate decision-makers about overall goal of care? (11)	○	○	○	○	○
Allowing trained social workers to provide emotional support to surrogates? (16)	○	○	○	○	○

4.8 What concerns would you have about this type of interprofessional surrogate support intervention that should be addressed in order for it to be acceptable?

____

____

____

____

____

4.9 Please include any thoughts about what strategies might be effective to improve the degree of interprofessional collaboration in ICUs for surrogates facing goal of care decisions.

____

____

End of Block: For Physicians / APPs only

Start of Block: NIH Questions

2.1 Because this study is funded by NIH we need to ask a few extra questions.

2.2 What is your gender?

- ○ Male (1)
- ○ Female (4)
- ○ Other (5)
- ○ Decline to answer (6)

2.3 What is your race?

- ○ White (1)
- ○ Black/African American (4)
- ○ Asian (5)
- ○ American Indian/Alaska Native (6)
- ○ Native Hawaiian/Pacific Islander (7)
- ○ Multiracial (8)

○ Decline to answer (9)

2.4 What is your ethnicity?

○ Hispanic/Latino (1)

○ Non-Hispanic/Latino   (4)

○ Decline to answer (5)

End of Block: NIH Questions

Start of Block: Vincent Payment Form

3.1 Thank you for your participation! Please complete the following information, so we may reimburse you for your time.

3.2 First Name:

____

3.3 Last Name:

____

3.4 Birthdate (DD/MM/YYYY):

____

3.5 SSN:
(You may refuse to give your SSN, but the University will have to reduce your payment by 24% for tax purposes)

____

3.6 Address:

- ○ Address (1) ____
- ○ City (2) ____
- ○ State (3) ____
- ○ Zip Code (4) ____

3.7 Phone Number:

____

3.8 Optional: Please enter your preferred 4 digit PIN code to use with your card:

____

End of Block: Vincent Payment Form

Start of Block: For All but Phy/APP

5.1 I feel confident in my ability to work with physicians and advanced practice providers to:

	Strongly disagree (1)	Disagree (2)	Neutral (3)	Agree (4)	Strongly agree (5)
Reinforce the prognostic information the physician provided to families (7)	○	○	○	○	○
Talk with surrogate decision-makers about	○	○	○	○	○

the various treatment options that the physician proposed (8)					
Elicit patients' values and preferences from surrogates facing goal of care decisions (1)	○	○	○	○	○
Discuss goal of care options with surrogates facing goal of care decisions (4)	○	○	○	○	○
Provide emotional support to surrogates (9)	○	○	○	○	○

5.2 I play an important role in providing support to patients' surrogates facing goal of care decisions.

- ○ Strongly disagree (1)
- ○ Disagree (2)
- ○ Neutral (3)
- ○ Agree (4)
- ○ Strongly Agree (5)

5.3 I would like to play a larger role in providing support to patients' surrogates facing goal of care decisions.

- ○ Strongly disagree (1)
- ○ Disagree (2)
- ○ Neutral (3)
- ○ Agree (4)
- ○ Strongly Agree (5)

5.4 I am encouraged by my colleagues to participate in supporting surrogate decision makers facing goal of care decisions.

- ○ Strongly disagree (1)
- ○ Disagree (2)
- ○ Neutral (3)
- ○ Agree (4)
- ○ Strongly Agree (5)

5.5 In general, I am made aware of scheduled goal of care meetings with families with enough advance notice that I can attend.

- ○ Strongly disagree (1)
- ○ Disagree (2)
- ○ Neutral (3)
- ○ Agree (4)
- ○ Strongly Agree (5)

5.6 I feel comfortable asking to be part of goal of care meetings.

- Strongly disagree (1)
- Disagree (2)
- Neutral (3)
- Agree (4)
- Strongly Agree (5)

5.7 I feel comfortable voicing any disagreement with support provided to surrogates facing goal of care decisions to physicians and/or advanced practice providers.

- Strongly disagree (1)
- Disagree (2)
- Neutral (3)
- Agree (4)
- Strongly Agree (5)

5.8 My role in supporting surrogate decision makers facing goal of care decisions is understood by other members of the healthcare team

- Strongly disagree (1)
- Disagree (2)
- Neutral (3)
- Agree (4)
- Strongly Agree (5)

5.9 My contributions in supporting surrogate decision makers facing goal of care decisions are valued by other members of the healthcare team

- ○ Strongly disagree (1)
- ○ Disagree (2)
- ○ Neutral (3)
- ○ Agree (4)
- ○ Strongly Agree (5)

5.11 I am well prepared to provide support to patients' surrogates facing goal of care decisions

- ○ Strongly disagree (1)
- ○ Disagree (2)
- ○ Neutral (3)
- ○ Agree (4)
- ○ Strongly Agree (5)

5.12 I would be interested in further training on communication skills for supporting surrogate decision makers facing goal of care decisions.

- ○ Strongly disagree (1)
- ○ Disagree (2)
- ○ Neutral (3)
- ○ Agree (4)

○ Strongly Agree (5)

End of Block: For All but Phy/APP

Supplement 2: Subset of Interview Questions Asked to 24 Participants (all types) at Sites without the PARTNER Program

1. One study looked to increase interprofessional support for surrogates by providing specialty communication training to nurses and instituting care pathways wherein these trained nurses checked in with families daily as well as helped families prepare, participate, and debrief after regularly scheduled family meetings—with the first occurring within 48 hours of enrollment into the intervention.
    a. What are your thoughts about this approach to achieving increased interprofessional collaboration in supporting surrogates?
    b. What might you imagine would be barriers to this approach?
    c. If you were to think about modifying this to work for your ICU, what would that look like?

eTable 1. General survey questions and responses

Question block for all participants (N=56)	Agree, n (%); median (IQR)[a]
3.2 Please indicate how much you agree with the following statements. In general, the healthcare team in my ICU:	
Establishes a high level of trust with surrogate decision-makers facing goal of care decisions.	49 (87.5%); 4 (4-5)
Effectively helps surrogates understand patient medical condition(s).	52 (92.9%); 4 (4-5)
Effectively helps surrogates understand patient prognoses (e.g. the likely outcomes of treatment).	43 (76.8%); 4 (4-4)
Effectively helps surrogates understand available treatment options as it relates to overall options in terms of goal of care (e.g., full life support, time-limited trial, and comfort-focused care).	50 (89.3%); 4 (4-5)
Effectively explores patient values and preferences as they relate to medical care.	39 (69.6%); 4 (3-5)
Effectively supports surrogates in applying patient values and preferences to decisions about overall goal of care.	42 (75%); 4 (4-5)
Promotes interprofessional collaboration between healthcare professions to support surrogate decision makers facing goal of care decisions.	44 (78.6%); 4 (4-5)
Achieves a high level of interprofessional collaboration between healthcare professions to support surrogate decision makers facing goal of care decisions.	41 (73.2%); 4 (3-5)
3.3 Higher levels of interprofessional collaboration between healthcare providers improves overall support provided to surrogate decision makers facing goal of care decisions.	55 (98.2%); 5 (5-5)
Question for Physicians/APPs only (N=20)[b]	Yes, n (%)
4.1 Are there any members of the ICU team that you would like to see have a higher degree of involvement in supporting surrogates facing goal of care decisions?<br>Yes, n (%)	10 (50%)
Question for Physicians/APPs only (N=20)[b]	n (%)
4.2 Please select those professions that you would like to see have a higher degree of involvement in supporting surrogates facing goal of care decisions<br>n (%)	
Registered Nurse	8 (40%)
Social Worker	8 (40%)
Care Manager	4 (20%)
Chaplain	7 (35%)
Other[c]	3 (15%)
Question block for ICU Physicians/APPs only (N=16-17)[d]	Comfortable, n (%); median (IQR)[e]
4.4 Currently, how comfortable would you be allowing:	
Nurses to reinforce the prognostic information you provided to families (they would not deliver new information, only restate what you conveyed)?	13 (81.3%); 4 (4-4)
Nurses to talk with surrogate decision-makers about the various treatment options that the physician proposed?	8 (50%); 4 (2-4)
Nurses to talk with surrogate decision-makers about patients' values and preferences?	17 (100%)[f]; 5 (4-5)
Nurses to talk with surrogate decision-makers about overall goal of care?	13 (81.3%); 4 (4-5)
Nurses to provide emotional support to surrogates?	16 (100%); 5 (5-5)

Social workers to reinforce the prognostic information you provided to families (they would not deliver new information, only restate what you conveyed)?	10 (62.5%); 4 (3-4)
Social workers to talk with surrogate decision-makers about the various treatment options that the physician proposed?	5 (31.3%); 3 (2-4)
Social workers to talk with surrogate decision-makers about patients' values and preferences?	15 (88.2%)[f]; 4 (4-5)
Social workers to talk with surrogate decision-makers about overall goal of care?	12 (75%); 4 (4-5)
Social workers to provide emotional support to surrogates?	16 (100%); 5 (5-5)
Question for ICU Physicians/APPs only (N=16)[d]	Supportive, n (%); median (IQR)[g]
4.5 We are considering developing an intervention that leverages multiple members of the ICU team (e.g., physicians/APPs, nurses, and social workers) to support family members making goals-of-care decisions for incapacitated, critically ill patients. The intervention would involve achieving shared agreement concerning who will support surrogates, what their respective roles will be, and how team members will work together in a well-integrated way. Additionally, the intervention would entail efforts to ensure that each team member has the needed expertise to effectively function as part of the team, and that there is trust and a shared mental model amongst all team members. Physicians and APPs would remain responsible for conveying prognostic information to surrogates, determining what are medically appropriate treatments to offer, and making treatment recommendations.	
4.6 If such an intervention were developed and shown to be effective, how supportive would you be of it being adopted in your ICU?	15 (93.8%); 4 (4-5)
Question block for ICU Physicians/APPs only (N=16-17)[d]	Comfortable, n (%); median (IQR)[e]
4.7 If members of your ICU team participated in this intervention to enhance interprofessional support to surrogates facing goals of care decisions, how comfortable would you be:	
Having trained nurses reinforce the prognostic information you provided to families (they would not deliver new information, only restate what you conveyed)?	15 (93.8%); 4 (4-5)
Allowing trained nurses to talk with surrogate decision-makers about the various treatment options that the physician proposed?	12 (75%); 4 (4-5)
Allowing trained nurses to talk with surrogate decision-makers about patients' values and preferences?	17 (100%)[f]; 4 (4-5)
Allowing trained nurses to talk with surrogate decision-makers about overall goal of care?	16 (94.1%)[f]; 4 (4-5)
Allowing trained nurses to provide emotional support to surrogates?	16 (100%); 5 (5-5)
Having trained social workers reinforce the prognostic information you provided to families (they would not deliver new information, only restate what you conveyed)?	13 (76.5%)[f]; 4 (4-5)
Allowing trained social workers to talk with surrogate decision-makers about the various treatment options that the physician proposed?	8 (50%); 4 (3-4)
Allowing trained social workers to talk with surrogate decision-makers about patients' values and preferences?	14 (82.4%)[f]; 4 (4-5)
Allowing trained social workers to talk with surrogate decision-makers about overall goal of care?	13 (76.5%)[f]; 4 (4-5)
Allowing trained social workers to provide emotional support to surrogates?	15 (93.8%); 5 (5-5)

Open ended response items for ICU Physicians/APPs only (N=16)[d]	
4.8 What concerns would you have about this type of interprofessional surrogate support intervention that should be addressed in order for it to be acceptable?	
• Nursing changes and sign-out per shift sometimes has led to miscommunication or communication breakdowns and discrepancies in the message being given by the team - would need addressed to consistency for plan to work.<br>• My concern is the availability of staff to provide this support and the high number of new nurses (and new graduates) that are joining the ICU.<br>• Certain aspects of treatment and clinical information should not be communicated by anyone other than physician or APP. The decision maker may ask questions that the nurse or social worker are not qualified to answer, or think they have the correct understanding when they do not.<br>• Ensuring non-physicians had a good understanding of medical and prognostic nuance and could appropriately convey that in a non-biased way.<br>• There would need to be very clear training about: - the boundaries of reinforcing what physicians had said instead of introducing new information (that may or may not be correct or agreed upon) - expected natural course of certain illnesses / care options (especially important for social workers who have less medical knowledge).<br>• Training is important but success depends more on the empathy and communication skill set of the individual practitioner.	
4.9 Please include any thoughts about what strategies might be effective to improve the degree of interprofessional collaboration in ICUs for surrogates facing goal of care decisions.	
• Education on the importance of communication regarding goals of care and providing designated times to have meetings with families.<br>• More involvement of palliative care APPs.<br>• More team-based training/ simulation.<br>• Well-seasoned caregivers would be the best fit for this approach.	
Question block for other healthcare professionals (N=31)	Agrees, n (%); median (IQR)[a]
5.1 I feel confident in my ability to work with physicians and advanced practice providers to:	
Reinforce the prognostic information the physician provided to families.	29 (93.5%); 4 (4-5)
Talk with surrogate decision-makers about the various treatment options that the physician proposed	28 (90.3%); 4 (4-5)
Elicit patients' values and preferences from surrogates facing goal of care decisions	28 (90.3%); 4 (4-5)
Discuss goal of care options with surrogates facing goal of care decisions	23 (74.2%); 4 (3-5)
Provide emotional support to surrogates	30 (96.8%); 4 (4-5)
5.2 I play an important role in providing support to patients' surrogates facing goal of care decisions.	29 (93.5%); 4 (4-5)
5.3 I would like to play a larger role in providing support to patients' surrogates facing goal of care decisions.	19 (61.3%); 4 (3-4)
5.4 I am encouraged by my colleagues to participate in supporting surrogate decision makers facing goal of care decisions.	22 (71%); 4 (3-4)
5.5 In general, I am made aware of scheduled goal of care meetings with families with enough advance notice that I can attend.	18 (58.1%); 4 (3-4)
5.6 I feel comfortable asking to be part of goal of care meetings.	26 (83.9%); 4 (4-5)
5.7 I feel comfortable voicing any disagreement with support provided to surrogates facing goal of care decisions to physicians and/or advanced practice providers.	19 (61.3%); 4 (3-4)
5.8 My role in supporting surrogate decision makers facing goal of care	24 (77.4%); 4 (4-5)

decisions is understood by other members of the healthcare team	
5.9 My contributions in supporting surrogate decision makers facing goal of care decisions are valued by other members of the healthcare team	25 (80.6%); 4 (4-5)
5.11 I am well prepared to provide support to patients' surrogates facing goal of care decisions	25 (80.6%); 4 (4-5)
5.12 I would be interested in further training on communication skills for supporting surrogate decision makers facing goal of care decisions.	30 (96.8%); 4 (4-5)

[a]'agree' includes both 'strongly agree' and 'agree'
[b]survey data missing for 5 participants
[c]included palliative care and respiratory therapy
[d]missing data from 1 ICU physician/APP and 1 other ICU physician/APP only completed a subset of items due to a branching logic error, resulting in N=16 or 17 depending on the item
[e]'comfortable' includes both 'very comfortable' and 'comfortable'
[f]items with N=17
[g]'supportive' includes 'moderately' to 'extremely supportive'

eTable 2. Demographics by clinician role (N=56)

Demographics	Physicians/APPs (N=25)[a]	Nurses (N=22)	Social workers (N=3)	Others (N=6)[b,c]
**Age, median (IQR), y**	40 (36-45)	29 (24-33)	38 (27-43)	63 (57-66)
**Gender, n (%)**				
Female	13 (54.2)	20 (90.9)	3 (100)	4 (66.7)
Male	11 (45.8)	2 (9.1)	0 (0)	2 (33.3)
**Race, n (%)**				
White	22 (91.6)	22 (100)	3 (100)	4 (66.6)
Asian	1 (4.2)	0 (0)	0 (0)	1 (16.7)
Decline to answer	1 (4.2)	0 (0)	0 (0)	1 (16.7)

[a]gender and race missing from 1 participant
[b]includes 1 care manager, 2 dieticians, and 3 spiritual care clinicians
[c]age missing from 1 participant

eTable 3. Clinicians' perceived barriers and facilitators to enhanced interprofessional support through specialty nurse training and care pathways

Determinant Category	CFIR Domain-Construct	Determinant	Description	Provider quote
**Barriers**				
	Inner setting-Work Infrastructure	Team turnover	Frequent changes in team members may limit rapport-building with surrogates.	"…I love the idea of the bedside nurse having some extra training and doing this…[but] the bedside nurse isn't the same person necessarily every day, and a lot of what we think about in palliative care is building rapport with families and surrogates." (Palliative clinician_4)
				"I think it would be hard for nurses, especially if the nurse is changing every so many days." (RN_4)
	Inner setting-Work Infrastructure and Opportunity	Time Constraints	Limited time team members have per shift given workload and competing responsibilities.	"…You can get pulled in a lot of different directions…so time constraints could be a barrier." (RN_3)
				"Nurses are exceptionally busy in the ICU, even with a two to one ratio…adding anything additional to their plate asks a lot…" (MD_4)
	Inner setting-Work Infrastructure	Evolving care plans	Challenges staying up to date and actively involved due to the dynamic nature of care planning in critical illness.	"…If that person's not fully engaged with all of the updates and all of the changes, it becomes hard for that person to be that communicator with all our information." (MD_3)
	Inner setting-Compatibility	Training Feasibility	Challenges in implementing training.	"[Training may] be tricky because you don't always have the ability to take staff away from the bedside for that long." (RN_4)
**Facilitators**				
	Inner setting-Access to Knowledge & Information	Specialized support training	Providing nurses with formal communication	"I think obviously like you said, [you need] training with the nurses… so they know how to

			and support training.	present information to the families." (APP_3)
	Innovation-Innovation Design	Clear role definition and consistent messaging	Explicitly defining each team member's responsibilities and ensuring consistent messaging to avoid introducing conflicting information.	"There would need to be very clear training about the boundaries of reinforcing what physicians said instead of introducing new information…" (MD_2)
				"[We would need to] make sure that we are on the same page." (MD_6)
	Inner setting-Work Infrastructure	Dedicated role	Having designated team members consistently responsible for surrogate support.	"I think you'd almost have to have a dedicated nurse to do that." (MD_4)
				"I think there has to be designated people [so that it's] not like a revolving door. There has to be key people who will be responsible for talking to surrogates on a daily basis and reporting it to the team." (Dietician_3)
	Innovation-Innovation Evidence-Base	Known evidence-base	Known effectiveness of interprofessional collaboration.	"If you tell a nurse right that we've done research and we found that if you do XYZ, that you'll be able to better support family. They would absolutely do it." (RN_4)
